# Gut Microbiome and Its Interaction with Immune System in Spondyloarthritis

**DOI:** 10.3390/microorganisms8111727

**Published:** 2020-11-04

**Authors:** Jacqueline So, Lai-Shan Tam

**Affiliations:** Department of Medicine and Therapeutics, Prince of Wales Hospital, The Chinese University of Hong Kong, Shatin, Hong Kong, China; so.jacqueline@gmail.com

**Keywords:** spondyloarthritis, gut microbiome, gut inflammation

## Abstract

Emerging evidence suggests there is a gut-joint axis in spondyloarthritis (SpA). In a study, subclinical gut inflammation occurred in nearly 50% of SpA. Chronic gut inflammation also correlated with disease activity in SpA. Trillions of microorganisms reside in the human gut and interact with the human immune system. Dysbiosis affects gut immune homeostasis and triggers different autoimmune diseases including SpA. The absence of arthritis in HLA-B27 germ-free mice and the development of arthritis after the introduction of commensal bacteria to HLA-B27 germ-free mice proved to be the important role of gut bacteria in shaping SpA, other than the genetic factor. The recent advance in gene sequencing technology promotes the identification of microorganisms. In this review, we highlighted current evidence supporting the link between gut and axial SpA (axSpA). We also summarized available findings of gut microbiota and its interaction with the immune system in axSpA. Future research may explore the way to modulate gut microorganisms in axSpA and bring gut microbiome discoveries towards application.

## 1. Introduction

Spondyloarthritis (SpA) is a common chronic inflammatory disease with a disease prevalence of 0.2% to 1.6% [1]. It mainly affects young people, with around 90% of them developing symptoms before 40 years old [2]. SpA is characterized by axial inflammation and peripheral manifestations including asymmetrical mono- or oligoarthritis, enthesitis and dactylitis. Patients often have extra-articular manifestations including psoriasis, uveitis and inflammatory bowel disease (IBD). According to the 2009 Assessment in Spondyloarthritis International Society (ASAS) classification, patients could be classified as axial SpA (axSpA) with predominant involvement of sacroiliac joint and/or spine or peripheral SpA with predominant peripheral manifestation [3]. AxSpA includes radiographic and non-radiographic SpA. Ankylosing spondylitis (AS) is the prototypic form of axSpA. Other subtypes of SpA include reactive arthritis, psoriatic arthritis, inflammatory bowel disease (IBD)-related SpA and undifferentiated SpA. To date, the cause of SpA is unknown.

The human gastrointestinal tract comprises up to 100 trillion bacterial microbes, which are exposed to the host through a mucus-covered surface area of 32 m^2^ [4,5]. Bacteroidetes and Firmicutes are the two major phyla, which account for nearly 90% of the microbes identified in the gastrointestinal tract, whereas Actinobacteria, Proteobacteria and Verrucomicrobia have a lower abundance [6]. At the genus level, *Bacteroides* species, *Faecalibacterium*, *Bifidobacterium*, *Lachnospiraceae*, *Roseburia* and *Alistipes* show a descending order of abundance [7]. Some microbes possess specific properties; for example, *Faecalibacterium prausnitzii* has anti-inflammatory effects [8]. Healthy individuals have high diversity concerning gut microbiome composition of Bacteriodetes, Firmicute, Actinobacteria, Spirochetes and Proteobacteria phyla. Reduction in microbiome diversity, reduction in beneficial bacteria and overgrowth of pathogenic microorganisms may trigger an uncontrolled immune response, leading to intestinal injury. Multiple factors including sex, comorbidities, diet, infection, antibiotic use, genetics, birth route, hygiene and stress affect the gut microbiome composition [9,10,11]. Intestinal dysbiosis, an imbalance of the microbiota, increases intestinal permeability. The exposure of a microorganism to the mucosal immune system triggers an immune response leading to different diseases [12].

Emerging evidence shows the link between gut and SpA. Around 5–7% of AS patients may develop IBD [13,14]. A large population study showed that the incidence of IBD was 5.3-fold higher in AS patients compared to healthy controls, whereas up to 13% of IBD patients developed AS, as was reported in a meta-analysis [15]. Around 50% of AS patients had subclinical gut inflammation in which chronic gut inflammation was associated with more extensive sacroiliac joint bone marrow edema [16,17]. Gut inflammation was also associated with increased risk of evolution from non-radiographic axSpA to AS and increased risk of IBD [18]. In this article, we focus on the current understanding of the relationship between gut and axSpA and explore the potential use of microbiota in treating axSpA.

## 2. Bowel Permeability and Intestinal Inflammation in Autoimmune Diseases

Luminal epithelium is important in maintaining normal homeostasis. It can absorb nutrients as well as act as a physical and biological barrier against microorganisms and antigens. Intestinal epithelium consists of six types of cells including absorptive enterocytes, goblet cells, Paneth cells, enteroendocrine cells, tuft cells and microfold villus cells. They secrete soluble factors such as mucin and anti-microbial peptides (AMP) including lysozymes, defensins and cathelicidin to prevent microbial invasion. Intercellular junctions between adjacent intestinal epithelial cells are formed by tight junctions, adherens junctions and desmosomes, which regulate the paracellular movement of ions, solutes and water across the epithelium [12]. Dysregulation of the interactions between luminal epithelial cells, immune cells and luminal microbiome increases bowel permeability, resulting in luminal antigen translocation which triggers immunological responses such as leukocyte recruitment and a release of soluble mediators, leading to intestinal inflammation [19]. Increasing evidence suggests that intestinal permeability is crucial in the pathogenesis of different autoimmune diseases including type I diabetes, celiac disease and IBD [12].

**Figure 1:**
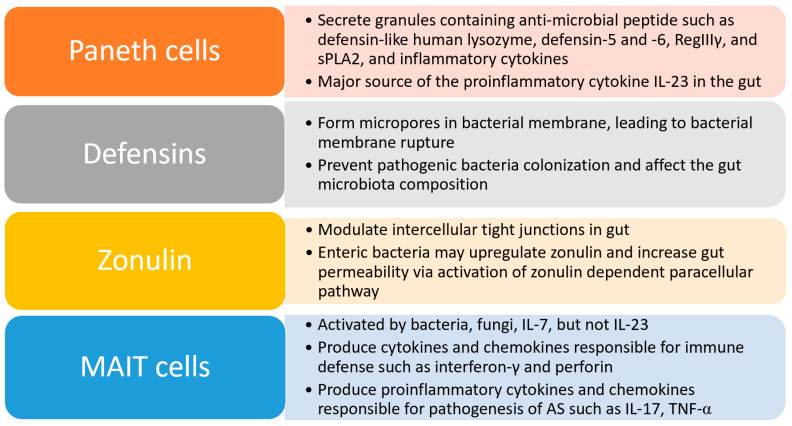
Key mechanisms in regulating gut barrier integrity.

Paneth cells are important in maintaining intestinal homeostasis by modulating microbial composition and regulating innate and adaptive immune responses (Figure 1). They secrete granules containing various anti-microbial peptides such as defensin-like human lysozyme, defensin(HD) -5 and -6, RegIIIγ, secretory phospholipase A2 (sPLA2) and inflammatory cytokines that affect intestinal inflammation, microbiota colonization and enteric pathogen invasion [20]. Paneth cells are also a major source of interleukin (IL)-23, a key proinflammatory cytokine, in both AS and Crohn’s disease (CD) patients [21].

Defensins are important AMP involved in the innate immunity in the intestinal mucosal barrier. Their productions are stimulated by exposure of pathological microbes to toll-like receptors and intracellular sensors. Defensin can form micropores in the bacterial membrane, resulting in an efflux of ions and water, and therefore bacterial membrane rupture. It can also prevent pathogen colonization. Lack of defensin due to nucleotide-binding oligomerization domain-containing protein 2 (NOD-2) mutation increases susceptibility to CD [22]. Reduction in α-defensin may affect luminal microbiota composition [23]. Upregulation of Paneth cell derived HD-5 was observed in terminal ileum of AS patients with acute intestinal inflammation and low inflamed ileum of CD patients. This suggests that overexpression of HD-5 and proinflammatory cytokines such as IL-23 may be involved in the pathogenesis of an early stage of AS and CD [21], followed by reduction in defensin, leading to bacterial translocation and inflammatory responses.

Zonulin, a modulator of intercellular tight junctions, is important in maintaining intestinal barrier permeability. Exposure of enteric bacteria to luminal epithelium can stimulate zonulin secretion. Genetic and environmental stimuli can also trigger dysregulation of the zonulin-dependent intestinal barrier, thus altering intestinal permeability [24]. Disruption of the epithelial barrier and dysbiosis can lead to an increase in both intestinal permeability and intestinal inflammation. A study showed that after exposure of the non-inflamed intestinal ileum of a CD patient to sodium caprate (a constitute of milk fat which can affect tight junctions), the non-inflamed tissue showed an increase in paracellular permeability with dilatations within tight junctions [25]. This showed luminal stimuli can alter mucosal permeability irrespective of gut inflammation. Damage of the gut vascular barrier, upregulation of zonulin and bacterial products were also observed in colonic tissue of AS patients [26].

Mucosal-associated invariant T (MAIT) cells are unique innate-like lymphocytes preferentially located in mucosal and epithelial barrier tissues, in particular gut lamina propria, and possess anti-bacterial activity [27]. Vitamin B2 (riboflavin) metabolites, produced by bacteria and fungi, can trigger the major histocompatibility class I-like antigen presenting molecule MR1 and activate MAIT cells. These trigger a rapid production of cytokines and chemokines responsible for host immune defense such as interferon-γ and perforin, as well as production of pro-inflammatory cytokines responsible for the pathogenesis of AS, including IL-17 and tumor necrosis factor (TNF)-α [27,28]. IL-7, produced by Paneth cells in the gut, can also stimulate MAIT cells to produce IL-17 in AS patients. Increased expression of IL-7 was also observed in both gut and synovial fluid of AS patients [29]. IL-23 is an important cytokine involved in the differentiation and maturation of T helper (Th)17 cells in AS. While there is a high expression of IL-23 receptor in AS patients, IL-23 priming of MAIT cells fails to stimulate the production of IL-17 [30]. The absence of MAIT cells in germ-free mice showed the essential role of gut commensal flora in the development and expansion of MAIT cells [31]. An elevation of MAIT cells was found in synovial joint fluid of AS patients, as well as in rheumatoid arthritis patients [32]. Disease activity in AS patients also correlated positively with MAIT cell activation. Similar to IBD patients, the level of blood MAIT cells in AS patients was lower than for healthy individuals, probably due to recruitment concerning inflamed sites such as gut and joint. However, there was an upregulation of IL-17 in MAIT cells in peripheral blood in AS patients. Taken together, the presence and activation of MAIT cells in both gut and joint of AS patients further support the link between gut and joint [30].

## 3. Key Cytokine Pathways in the Pathogenesis of Inflammatory Bowel Disease and Spondyloarthritis

**Figure 2:**
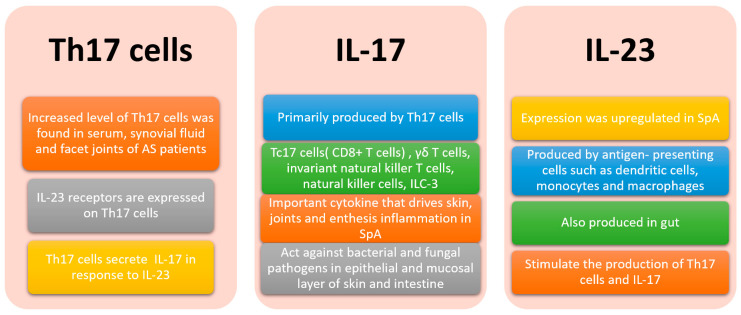
The role of IL17/IL23 axis in spondyloarthritis (SpA).

The “gut-synovial axis” was hypothesized in view of the link observed between SpA and IBD. Various host and environmental factors, including gut dysbiosis, genetic predisposition, infection and diet, trigger a cascade of immune responses leading to autoimmune diseases. The IL-23/ IL-17 axis is believed to be crucial in the pathogenic mechanism in axSpA and IBD (Figure 2) [33]. IL-17 promotes T cell priming and stimulates fibroblasts, endothelial and epithelial cells and immune cells such as macrophages, to produce pro-inflammatory cytokines and chemokines [34]. IL-17A and IL-17F also stimulate the production of anti-microbial peptides β-defensins at the epithelial layer which is important for maintaining gut permeability [35]. Th17 cells are the main source of IL-17. Tc17 cells (CD8+ T cells), γδ T cells, invariant natural killer T cells, natural killer cells and type 3 innate lymphoid cells (ILC-3) also produce IL-17 [35]. Other pro-inflammatory cytokines produced by Th17 cells include IL-6, IL-22, IL-26, interferon-γ and TNF-α. IL-23 plays a major role in activation of T cells, which results in expansion of Th17 cells.

The B27- transgenic rat model showed that Th17 cells play an important role in the development of SpA through induction of proinflammatory cytokines such as IL-17 and TNF-α [36]. In B27/HuB2m- transgenic rats with spondyloarthritis-like disease, the IL-23/IL-17 axis was strongly activated and was associated with colonic inflammation. IL-23 promotes the development of colitis through upregulating downstream proinflammatory meditators such as IL-17, IL-1, IL-6 and TNF in a rat model [37].

The serum levels of IL-17 and IL-23 were raised in AS patients compared to healthy control [38]. An abundance of IL-17-secreting cells in the facet joint was also noted in AS [39]. Similar to the animal studies, upregulation of IL-23 was found in terminal ileum of AS and CD patients. However, upregulation of IL-17 was only observed in the gut of CD but not in AS patients [40].

In a human clinical trial, anti-IL-17 such as secukinumab and ixekizumab were effective at treating AS. However, anti-IL17 may worsen gut inflammation in AS patients with IBD. IL-17 is important in maintaining intestinal epithelium barrier integrity by repairing damage epithelium and avoiding overgrowth of bacteria that promote gut inflammation [41]. In IL-17-deficient mice, dysregulated gut permeability and atypical distribution of epithelial tight junction protein occlusion were observed [42]. Blocking of upstream of Th17 cells by anti-IL-23 such as Risankizumab and Ustekinumab, which are useful in treating IBD, failed to demonstrate efficacy in treating AS. Possible hypotheses include IL-23-independent induction of IL-17 from immune cells and the presence of other key regulatory agents other than IL-23 in targeting a Th17 response in AS [43].

Upregulation of IL-32 was observed in both CD and AS patients with chronic intestinal inflammation [44]. IL-32 overexpression was accompanied with an increase in proinflammatory cytokines such as IL-1B, IFN-γ and TNF-α in CD, but not AS. Only IL-10 was strongly correlated with IL-32 in AS, in which IL-10 is crucial in maintaining epithelial permeability [45]. Moreover, IL-22 was also overexpressed in ileum of AS patients, which was accompanied with an increase in IL-23 but not IL-17 [46]. IL-22 is released by the mucosal natural killer cell NKp44+. Increased expression of IL-22 is protective against ileitis in the animal model [47]. In the absence of IL-17, IL-22 can protect gut mucosa from inflammation by promoting goblet cell hyperplasia and mucin production [46,48]. These suggest the tissue-protective role of IL-32 and IL-22 in gut in AS patients.

IL-17A and IL-17F are important for protecting against cutaneous infection with *Candida albicans* and *Staphylococcus aureus* and intestinal infection by *Citrobacter rodentium* as shown in animal studies [35]. Segmented filamentous bacteria (SFB) are gram-positive bacteria that selectively colonize in terminal ileum. SFB induce expression of Th17-associated genes (IL-17, IL21, Cer6, NOS2) and anti-microbial gene RegIIIγ. Colonization with SFB alone in mice intestine is sufficient for the differentiation of Th17 cells in lamina propria, which produce IL-17 and IL-22, and protect mucosal from *Citrobacter rodentium* infection [49]. Commensal bacteria promote epithelial fucosylation by prompting ILC3 to produce IL-22 [50]. Fucosylation of intestinal epithelial cells liberates fucose into the lumen and its metabolism is also affected by intestinal bacteria [51]. This process reduces the expression of bacterial virulence genes, prevents colonization of intestinal opportunistic bacterium *Enterococcus faecalis* and enhances tolerance of harmful bacteria such as *Citrobacter rodentium* [52].

## 4. Genetic Predisposition to the Development of Gut Inflammation and Spondyloarthritis

The genetic factor plays an important role in the pathogenesis of SpA. HLA -B27 is an allele of the HLA-B locus in the class I region of the human major histocompatibility complex (MHC). The association between HLA-B27 and the development of AS was the strongest between an MHC antigen and a disease, with over 90% of the AS patients being HLA-B27 positive [2]. However, only 5% of the HLA-B27 carrier will develop AS. Misfolding of HLA-B27 promotes endoplasmic reticulum stress and triggers unfolded protein response, which in turn stimulates IL-23 production and bowel inflammation [53].

Genome-wide association studies found that AS and IBD patients shared over 10% of their gene pathways, in which genes involved in the Th17 cell pathway are of significant importance, including IL-23R, IL-12B, STAT3 and caspase recruitment domain-containing protein 9 (CARD9) [54,55,56]. STAT3 is a major signaling molecule within the Th17 lymphocyte differentiation pathway. IL-23 signals through its receptor IL-23R and induces STAT3 phosphorylation [57]. This stimulates Th17 cells to produce proinflammatory cytokines such as TNF, IL-1B and IL-17, leading to joint and bowel inflammation [54]. CARD9 is an adaptor protein highly expressed in dendritic cells and in macrophages which can regulate the innate immune response to selected intracellular bacteria, fungi and viruses. It stimulates Th17 cell differentiation, IL-23, TNF-α and other cytokines’ production, resulting in joint and gut inflammation [33]. IL-12B encodes IL-12p40, which is the common subunit of IL-23 and IL-12. IL-23 mediates chronic inflammation and IL-12 promotes naïve T cell differentiation. IL-12B genetic polymorphism confers susceptibility to AS and is associated with disease severity [58].

NOD2, previously known as caspase recruitment domain-containing protein 15 (CARD15), is an intracellular protein encoded by the NOD2 gene. The NOD2 variant is associated with CD and predisposes CD patients to sacroiliitis [59,60,61]. However, NOD2 polymorphism does not confer AS susceptibility [60].

## 5. Dysbiosis and Spondyloarthritis

The mouse model showed the link between gut microbiota and SpA. BALB/c ZAP-70^W163C^ mutated (SKG) mice increase thymic production of arthritogenic autoimmune T cells and result in IL-17-dependent SpA-like inflammatory arthritis (Table 1). SKG mutated mice developed inflammatory arthritis in conventional microbial conditions, but remain healthy in specific pathogen-free (SPF) conditions [62]. β-glucan is a major component of fungal and some bacterial cell walls which can trigger the dectin-1 receptor in synovial cells leading to synovitis [63]. After a systemic injection of curdlan, 1,3-β-glucan aggregated to SPF SKG mutant mice, and all developed inflammatory arthritis and more than half of them developed small intestine inflammation, with features similar to those in CD [64]. Expression of tight junction protein occludin in ileum was significantly reduced in curdlan-treated SPF SKG mice, as compared to naïve SPF SKG mice [62]. Moreover, curdlan-treated SKG mice had higher incidence of arthritis and more severe ileitis as compared to germ-free SKG mice. Moreover, injection of mannan, another microbial cell wall component, can also induce peripheral arthritis in SKG mice, but not ileitis [64]. Together, these suggest multiple microbial cell wall components could trigger SpA-like disease in SKG mice independently.

Despite the high genetic predisposition of HLA-B27, the concordance rate in the homozygotic twin is only 50–70%, which signifies that other environmental factors may also play a role in the pathogenesis. Animal models showed that the gut microbiome is essential in the development of AS. None of the HLA-B27 germ-free mice developed AS [65]. Interestingly, after the introduction of common luminal bacteria, over 80% of them developed AS and diarrhea [66]. Different gut microbiome compositions also affect the degree of gut inflammation in AS patients; in particular, *Bacteroides specie* was found to be associated with intestinal inflammation in B27 transgenic rats [66]. The presence of HLA-B27 and a different genetic background can also alter gut microbiome composition in AS [67,68]. Increased abundance of bacteria that promote gut inflammation including *Akkermansia muciniphila* and *Prevotella* were observed in the Fischer and Lewis strain B27 transgenic rats, respectively. *Akkermansia muciniphila* can degrade mucin in gut epithelium and *Prevotella* can enhance Th17-mediated immune responses in gut mucosa [69,70].

HLA-B27 expression can also affect intestinal metabolome. In the B27-transgenic rat model, the administration of microbial metabolite propionate significantly reduced the production of proinflammatory cytokines including IL-1B, IL-17A and IFN-γ and colonic inflammation [71]. An increase in *Prevotella* *specie* and a decrease in *Rikenellaceae* relative abundance were observed concerning HLA-B27 transgenic animals, compared to wild type rats. The abundance of *Bacteroides vulgatus* was augmented in HLA-B27/hβ2m and hβ2m compared to wild type rats. This showed HLA-B27 may alter the gut microbiome composition [72].

Animal models also showed that gut microbiota can promote Th17 cell differentiation, a key driver of SpA, in lamina propria of the small intestine [73]. Th17 cells were absent in lamina propria in germ-free K/BxN mice. Delayed onset of arthritis and less severe arthritis were also observed in germ-free K/BxN mice. After the introduction of segmented filamentous bacteria, gram-positive, spore-forming obligate anaerobes, to germ-free K/BxN mice, the expression of Th17 cells in lamina propria and IL-17 production increased and the development of arthritis was accelerated [49,74]. IL-23 also alter microbiome composition in AS. In SKG mice that develop IL-23-dependent SpA-like arthritis, the introduction of anti-IL-23 decreased *Bacteroidaceae*, *Porphyromonadaceae* and *Prevotellaceae* and increased *Clostridiaceae* and *Lachnospiraceae* abundance [75].

Earlier studies proposed a role for *Klebsiella* in the development of AS, evidenced by the isolation of *Klebsiella* from faecal cultures and detection of an anti-*Klebsiella* antibody in AS patients [76]. However, the association between *Klebsiella* and AS is weak. Moreover, there was no significant oral microbiome difference observed in SpA patients compared to healthy control [77].

Dysbiosis is associated with disease activity in AS patients, evidenced by the positive correlation between the abundance of the genus *Dialister* in colonic biopsies and the AS Disease Activity Score (ASDAS) [78] and the positive correlation between the abundance of *Ruminococcus gnavus* in faeces and the Bath Ankylosing Spondylitis Disease Activity Index (BASDAI) [79]. Dysbiosis is also associated with gut inflammation in AS patients [26,80]. Terminal ileum biopsies of biologic naïve AS patients showed a strong microbial imbalance as compared to healthy controls, with increased abundance of five families of bacteria including *Lachnospiraceae*, *Ruminococcaceae*, *Rikenellaceae*, *Porphyromonadaceae* and *Bacteroidaceae* and a decrease in the abundance of two families of bacteria—*Veillonellaceae* and *Prevotellaceae* [81]. Studies showed a distinct faecal microbiota pattern in AS patients; however, the results are inconsistent. A higher abundance of *Bifidobacterium* and *Prevotellaceae*, including *Prevotella melaninogenica, Prevotella copri* and *Prevotella* species, was observed in faecal samples of Chinese AS patients, as compared to healthy controls [82]. Another study conducted in Sweden showed higher abundance of *Proteobacteria*, *Enterobacteriaceae*, *Bacilli*, *Streptococcus species* and *Actinobacteria* in faecal samples of AS patients when compared to healthy control, but lower abundance of *Bacteroides* and *Lachnospiraceae* [80]. A study in France found disease-specific dysbiosis in SpA patients with higher abundance of *Ruminococcus gnavus* in faecal samples [79]. However, the difference in *Ruminococcus gnavus* abundance in faecal samples of SpA patients was not observed in another study [83]. A reduced abundance of *Bacteroides* was found in SpA patients, but not in *B. fragilis*. A trend of decreased abundance of *Faecalibacterium prausnitzii*, which demonstrated strong anti-inflammatory effects both in vitro and in vivo, was observed in faecal samples of SpA patients [80,83,84] (Table 2).

Changes in microbiome composition were also observed in pre- and post-biologic treatment in SpA patients. In a study of SpA and PsA patients (SpA/PsA), distinctive microbiome signatures were observed in this cohort with increased abundance of Clostridiales and Erysipelotrichales order and lower abundance of Bacteroidales order, when compared to healthy individuals [85]. An increase in Clostridiales and a reduction in Bacteroidales abundance were observed in this cohort after anti-TNF treatment. Interestingly, reverse abundance with lower abundance of Clostridiales and increase abundance of Bacteroidales were observed post-anti-IL17 treatment.

Perturbation of mycobiome was also observed in this SpA/PsA cohort after biologic treatment. An increase in the abundance of fungal taxa Saccharomycetales order was observed in SpA/PsA patients after anti-TNF and anti-IL17 [85]. *Candida* and *C. Albicans* expanders before biologic treatment were associated with higher abundance of *Bacteroides* post treatment. Another pilot study also showed characteristic gut mycobiome in AS patients with an increased abundance of Ascomycota at the taxonomic level, especially for the class of Dothideomycetes, and decreased abundance of Basidiomycota, especially for Agaricales [86].

Current studies did not give a conclusive finding of a SpA-specific gut microbiome or mycobiome pattern. The difference in the outcomes of various studies may be explained by host factors, environmental factors and technical variations such as DNA extraction protocol and choice of PCR primer. With the knowledge of these factors, future studies with better study designs could be conducted in order to identify disease-specific gut microbiome.

## 6. Potential Treatment: Antibiotic, Probiotic and Faecal Microbiota Transplantation

Currently, non-steroidal anti-inflammatory drugs and biologics including anti-TNF and anti-IL-17 are the mainstay of treatment for axSpA. With the growing evidence supporting the link between gut and SpA, novel treatments that could modulate gut microbiota such as antibiotics and probiotics are being investigated.

Sulphasalazine is mainly composed of salicylic acid and an antibiotic, sulfapyridine. It is effective in treating both peripheral SpA and IBD. A decrease in non-spore forming anaerobes was observed in IBD patients after taking sulphasalazine. A resolution of gut inflammation and joint improvement after sulphasalazine is observed in seronegative SpA [87]. Moxifloxacin is a fluroquinolone group antibiotic that acts against some gram-positive and -negative bacteria and exhibits immunomodulatory effects. It can inhibit proinflammatory cytokines IL-1 and TNF-α synthesis. In an open labelled pilot study, moxifloxacin showed significant improvement in disease activity and inflammatory markers in AS patients [88]. Another antibiotic, Rifaximin, was effective in preventing AS progression and modulating gut microbiota composition in the mouse model [89]. However, these results were mainly based on trials with a small sample size and animal models. Larger clinical trials are required to validate the efficacy of antibiotics in treating SpA patients.

In view of the complexity and the dynamic changes of gut microbiome, future animal models and human studies should be performed in order to better understand the bacterial taxonomics and their functions in gut in SpA patients and how gut microbiome arises and evolves, such that culprit pathogens involved in triggering SpA could be identified. This may shed light on potential disease-specific antibiotic treatment in treating SpA.

Probiotics are a combination of beneficial live bacteria and yeast. Prebiotics are fibres that promote growth of selected bacteria. In HLA-B27 transgenic rats that develop colitis, gastritis and systemic inflammation, *Lactobacillus rhamnosus* is effective in preventing colitis [90]. Prebiotic treatment is also effective in reducing colitis in HLA-B27 transgenic rats [91]. These suggest a potential role for probiotic and prebiotic modulating of the disease. However, in a randomized controlled trial, oral probiotic was not effective in treating SpA [92]. Future clinical studies are warranted to identify beneficial strains of bacteria and thus the optimal probiotic/prebiotic formula that could modulate the gut flora and ultimately treat SpA.

Faecal microbiota transplantation (FMT) aims to restore gut homeostasis by transferring gut bacteria and microbes from healthy individuals’ feaces. It is highly effective in treating refractory and recurrent *Clostridium difficile* infection. There is growing popularity in studying the use of FMT in different diseases including IBD, metabolic diseases and other autoimmune diseases including axSpA. Currently, there is an ongoing double-blinded placebo controlled randomized pilot study comparing the use of FMT and placebo in treating active axSpA, we are hoping for promising results [93].

Another potential role of the study of gut microbiome is to identify gut bacteria that can be used as biomarkers to predict the therapeutic efficacy of biologicsand guide personalized treatment in SpA.

## 7. Conclusions

Current literatures clearly demonstrates the link between gut microbiome and its interaction with the immune system in SpA. The reality of the essential role of gut microbiome in the pathogenesis of SpA is supported by animal models and some human studies. Future studies are required to identify the core microbiome associated with SpA, which thus might be a promising therapeutic target for treatment of axSpA. With a better understanding of the ecosystem in gut in axSpA, potential therapeutic agents such as antibiotic, probiotic, prebiotic and FMT may help restore a healthy gut microbiome in axSpA by precision microbiome manipulation, and hopefully make axSpA into a cure.

## Figures and Tables

**Table 1:** Murine Models of SpA Associated with Dysbiosis.

Mice Strain	Environmental Condition	Reference
SKG	◾ GF: no arthritis ◾ Conventional: arthritis	[54]
SKG	◾ GF, curdlan: arthritis ◾ SPF, curdlan: arthritis ◾ GF, recolonized with ASF, curdlan: arthritis (less severe than SPF, curdlan)	[54]
SKG	◾ SPF: no arthritis ◾ Conventional: all develop arthritis ◾ Transfer of thymocytes/splenocytes from SPF SKG to BALB/c athymic nude mice: severe arthritis	[55]
SKG	◾ SPF, Zymosan: arthritis ◾ Conventional, amphotericin B: no arthritis ◾ SPF, Zymosan, amphotericin B: arthritis ◾ SPF, curdlan or laminarin, SKG: chronic arthritis ◾ SPF, curdlan or laminarin, BALB/c: transient arthritis	[55]
SKG	◾ SPF: no arthritis ◾ SPF, curdlan, 1,3-β-glucan: all develop arthritis, 40–50% developed dactylitis, 50–60% developed small intestine inflammation, 25% developed acute unilateral uveitis	[56]
B27 transgenic rat	● GF: no peripheral arthritis/gut inflammation ● Conventional: 80% developed arthritis and colitis ● Different genetic background: affect gut microbiome composition	[57,58,59,60]

SKG: BALB/c ZAP-70W163C mutated. GF: Germ free. SPF: Specific pathogen-free. Zymosan includes β-glucans and mannan, which are key components of yeast cell walls. Purified β-glucans: Curdlan and laminarin.

**Table 2:** Altered Gut Microbiota in Faecal Samples in SpA Compared to Healthy Individuals in Animal and Human Studies.

	Increased Abundance	Decreased Abundance	Reference
HLA-B27/hβ2m, compared to wild type	*Prevotella* spp.	*Rikenellaceae*	[72]
HLA-B27/hβ2m and hβ2m, compared to wild type rats	*Bacteroides vulgatus*		[72]
SKG mice+ anti-IL23	*Clostridiaceae* and *Lachnospiraceae*	*Bacteroidaceae, Porphyromonadaceae* and *Prevotellaceae*	[75]
HLAB27 positive individual (in the absence of disease or treatment)	*Roseburia* species at left colon, right colon, rectum, and faeces *Neisseriaceae* at cecum and ileum	*Bacterioides ovatus* across multiple sites (ileum, cecum, left colon, right colon, and faeces) *Blautia obeum* at left colon and right colon *Dorea formicigenerans* at rectum and faeces	[68]
SpA patients, compared to RA and healthy controls	Ruminococcus gnavus		[79]
Chinese AS patients, compared to healthy control	*Bifidobacterium* and *Prevotellaceae* including *Prevotella melaninogenica*, *Prevotella copri* and *Prevotella* species		[82]
Sweden AS patients, compared to healthy control	*Proteobacteria, Enterobacteriaceae, Bacilli,* *Streptococcus species, Actinobacteria Bacteroides* and *Lachnospiraceae*		[80]
Biologic naïve PsA/SpA patients	Clostridiales and Erysipelotrichales order	Bacteroidales order	[85]
PsA/SpA patients	After anti-TNF: Clostridiales After anti- IL17: Bacteroidales After anti-IL17 and anti-TNF: Saccharomycetales order	After anti-TNF: Bacteroidales After anti-IL17: Clostridiales	[85]
AS patients	Ascomycota at taxonomic level, especially the class of Dothideomycetes	Basidiomycota, especially Agaricales	[86]
